# Developing a Discrete Choice Experiment Questionnaire to Design Health Policy Interventions for Rural Retention of Specialist Physicians in Rajasthan, India

**DOI:** 10.7759/cureus.76073

**Published:** 2024-12-20

**Authors:** Anushree Joshi, Jallavi Panchamia, Dileep Mavalankar

**Affiliations:** 1 Department of Health Policy, Management and Behavioural Science, Indian Institute of Public Health Gandhinagar, Gandhinagar, IND

**Keywords:** choice sets, discrete choice experiment questionnaire, experimental designs, rural healthcare system, specialist physicians

## Abstract

Background: Understanding the preferences of specialist physicians is essential to mitigate their critical deficiency in the Indian rural healthcare system. This necessitates an urgent focus to inform health policy interventions imperative to address and strengthen the vacancies of specialist physicians in the Indian rural healthcare system. The policy interventions should address the preferences of specialists, leading to their intention to stay in rural postings. The paper aims to develop a questionnaire to assess specialist physicians' preferences for rural postings using a discrete choice experiment (DCE).

Materials and methods: A DCE is a widely utilized quantitative approach to understanding health workers' preferences, positing that individuals make trade-offs while selecting an alternative product or service that provides the most utility. This paper comprehensively explains the stages of developing the DCE questionnaire, which involves creating choice sets using various experimental designs to ascertain specialist physicians' preferences for rural postings.

Results: The choice sets for the specified attributes and levels in the study were generated by mathematically combining hypothetical job scenarios using diverse experimental designs. The study employed a pairwise design for the mathematical combination of hypothetical job scenarios, yielding 90 unique choice sets with an equal likelihood of involvement across each of the six blocks. Consequently, each of the six blocks contained 15 distinct choice sets, administered to participants as six DCE questionnaire versions.

Conclusion: This paper outlines the creation of a DCE questionnaire aimed at elucidating the incentive preferences of specialist physicians in rural Rajasthan. It describes the development of diverse experimental designs and the creation of choice sets for the questionnaire's formulation. The objective is to offer a comprehensive guide for novice researchers, doctoral scholars, and health practitioners, imparting information and comprehending the intricacies involved in DCE questionnaire design, even if they are new to this research methodology.

## Introduction

An appropriate healthcare workforce is essential for an adequately functioning healthcare system [1]. The disparity in the dispersion of healthcare workers, especially in rural areas of India, is a significant issue in strengthening the country's rural healthcare system [2]. Due to numerous benefits, healthcare professionals frequently opt to work in metropolitan areas rather than in underserved locations [3-6]. Urban locations offer innumerable benefits, including enhanced income prospects, increased productivity facilitated by a better quality of equipment and accommodation facilities, a safer work environment, and higher professional growth opportunities compared to their rural counterparts [3-6]. Private health facilities, the leading providers of specialized treatments, are predominantly in urban areas [3-6]. As a consequence, the resulting limited access to health providers in rural regions has considerable repercussions for the deaths of mothers and children and various communicable diseases, leading to a higher burden of disease and mortality in Indian rural communities [3,4].

India's rural healthcare system is currently encountering a significant deficiency of specialist physicians at rural community health centres (CHCs) - thirty-bed, round-the-clock rural hospitals catering to about 120,000 individuals, with primary health centres (PHCs) at the top and health sub-centres (SCs) at the bottom [7-9]. There are 165,639 SCs, 25,354 PHCs, and 5,491 CHCs in the rural areas of the country, compared with 3,976 SCs, 6,528 PHCs, and 868 CHCs in the urban areas of the country [7,9].

As per the Indian Public Health Standards norms, the CHCs must be staffed by four specialist physicians: a surgeon, a physician, a paediatrician, an obstetrician, and a gynaecologist, along with supporting staff [7]. With a facility comprising 30 inpatient beds, CHCs are supposed to offer specialized care in general medicine, obstetrics and gynaecology, surgery, and paediatrics [7,9]. Specialist physicians (or specialists) refer to clinicians who have completed higher training in a specialized field of medicine. They offer specialized services to rural communities through a staff of healthcare workers at rural CHCs [7,9].

According to the Health Dynamics of India (Infrastructure and Human Resources) 2022-2023 Report, rural CHCs have a 79.5% deficit of specialist physicians compared to urban CHCs, which face a 58.7% deficit of specialist physicians in the country [9]. There occurs a deficit of 54.8% of physicians, 61.8% of surgeons, 52.3% of paediatricians, and 58.7% of obstetricians and gynaecologists (OB-GYNs) in the urban CHCs of the country [9]. However, the issue is more prominent among the country's rural regions, as 64.8% of the population lives in rural areas compared to 35% of the population in urban areas [9]. The country's increasing rural healthcare system needs 69.2% of physicians, 83.2% of surgeons, 67.1% of paediatricians, and 74.2% of OB-GYNs to improve the availability and management of vital rural healthcare services [9]. Only 913 of India's 5,491 functioning rural CHCs have all four required specialists in them, which calls for addressing the critical need for more specialist physicians in rural regions of the country [9]. Various rural retention regulatory measures were implemented in the past under the National Rural Health Mission launched in 2005 by the Government of India [8]. These included providing financial incentives, improving working conditions, rotational postings in the rural regions, an increased number of sanctioned posts, locality-specific recruitment, and a new service cadre specifically for public sector employment [8]. However, despite these initiatives, challenges persist, calling for an evidence-based approach to understanding this long-standing shortage of health professionals in rural regions [8].

An adaptable and efficient healthcare system considers the preferences and requirements of its healthcare professionals [1,2]. Building an effective healthcare system depends on motivated healthcare professionals, whose opinions and personal preferences are integral to functioning [1,2]. Therefore, it is crucial to comprehend the preferences of specialist physicians to design job incentive packages to enhance their job satisfaction. When presented with the necessity to figure out among multiple scenarios and make trade-off decisions, discrete choice experiments (DCEs) are valuable tools for determining health workers’ preferences [10]. DCEs are a quantitative approach for assessing the value of various job factors affecting the employment decisions of health workers [10-12]. This method offers quantitative insights into the relative significance of multiple job characteristics that impact health workers' employment choices and the likelihood of job acceptance [10-12]. As evident in the literature, the DCE method has been employed to solve retention challenges of healthcare professionals both in developed countries and low- and middle-income countries (LMICs) [10-13]. The objective of the current study is to offer a detailed explanation for designing a DCE questionnaire to enhance the rural retention of specialist physicians in Rajasthan, India, which involves the creation of various choice sets (paired hypothetical job scenarios) using different experimental designs. Using this DCE questionnaire, the aim is to understand the incentive preferences of specialist physicians in Rajasthan, India - one of the largest Indian states currently facing an acute shortage of specialist physicians and inform policy interventions [14,15]. Our research paper provides a detailed explanation of the mechanism of constructing a DCE questionnaire using choice sets employing different experimental designs. This can benefit novice researchers, doctoral scholars, and health practitioners seeking comprehensive knowledge of this research methodology.

## Materials and methods

Study settings

Rajasthan, with a population of 68.6 million, experiences a shortage of health human resources (HHR), particularly among specialists in rural areas, which comprise 75.13% of the state’s population [14-16]. There remains a deficit of 2090 specialists relative to the sanctioned capacity. To enhance the retention of specialist physicians and the availability of vital healthcare services in the rural healthcare system, there remains a requirement for 650 physicians, 650 surgeons, 650 OB-GYNs, and 650 paediatricians [9]. 

Study design: discrete choice experiment

The study employed a distinctive approach - DCE-integrating qualitative and quantitative approaches to evaluate specialist physicians' incentive choices and motivations to practice in rural CHCs in Rajasthan. It was conducted from September 2021 to August 2022, following the IIPHG Institution Ethics Committee’s approval. DCE is based on Lancaster's theory of value (1966) [17], which posits that each good may be represented by a combination of features and their respective degrees. Based on the random utility framework [18], this method uses statistical design theory to create sets of choices that describe different policy options. After that, participants will select their most liked alternative from these sets of choices. DCEs depend on participants' stated intentions, known as stated preference data, rather than their actual behaviours, termed revealed preference data [17,18].

Phase 1: identification of job factors

DCE's questionnaire development employs several methodologies to delineate attributes, including literature studies, theoretical frameworks, current health outcome measures, in-depth interviews, and focus group discussions [19,20]. For our study, the first step of the questionnaire development involved the extraction of job attributes and their associated levels by exploring the relevant HHR literature, followed by a few informal telephonic interviews with the key informants (KIs) [10,12]. This was useful in getting an idea about the rural posting and retention challenges of specialists and helped us develop an interview guide. Using a purposive sampling technique, 21 participants were telephonically interviewed with the help of an interview guide to determine the job attributes and their corresponding levels. These 21 participants comprised second- and third-year medical residents pursuing their post-graduation and specialist physicians selected from public health facilities of Rajasthan in the disciplines of paediatrics, surgery, medicine, obstetrics, and gynaecology [9,15,16]. The average duration for the interview was one hour. The 11th interview achieved data saturation; however, more interviews were conducted to obtain deeper insights. The identified job attributes included factors such as salary, rural retention bonus, staffing level and workload, transfer and promotion policy, residential facilities, workplace infrastructure, and workplace location. This qualitative phase served as an essential step in guiding the subsequent phase of the study, which involved the development of different experimental designs and the creation of choice sets [12].

Phase 2: construction of different experimental designs

**Figure 1:**
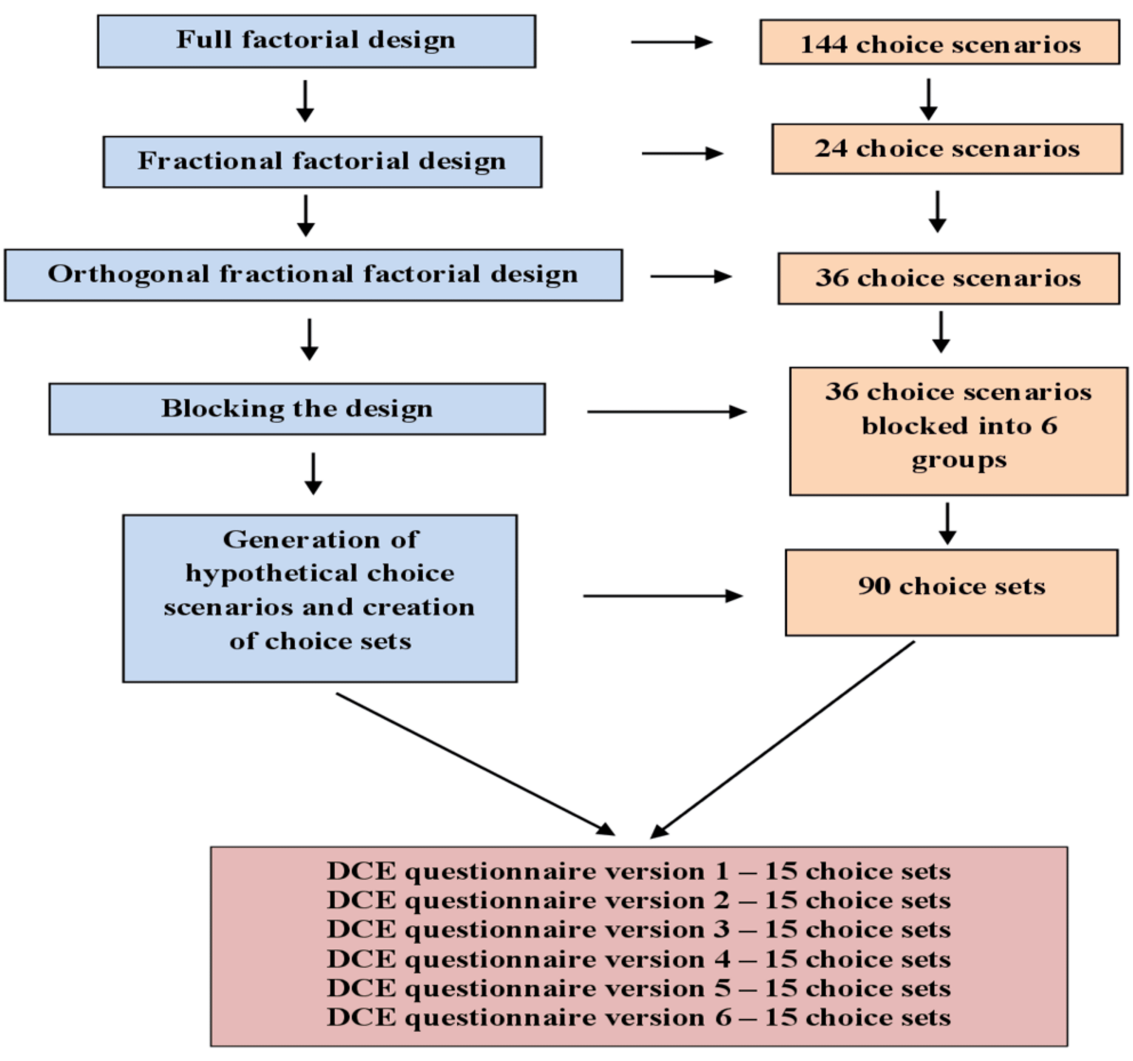
Stages of DCE questionnaire development using various experimental designs DCE: discrete choice experiment

The second step of the DCE questionnaire development comprised generating various experimental designs [12]. Considering the multiple arrangements of the six job factors and their associated levels, different experimental designs were generated to create choice sets for the DCE questionnaire. In performing a DCE, it is advisable to maintain an optimal number of attributes between five and eight since an excessive number can overwhelm participants and complicate the analysis [10-13]. The selection of these six attributes and their corresponding levels was determined using a ranking approach based on their ascending importance as assessed by the participants [10-13]. Ngene statistical software (version 1.3) [21] was used to construct different experimental designs using the specific syntaxes to optimize D-efficiency (a measure employed in experimental design to assess the efficacy of a specific design in estimating treatment effects; a higher D-efficiency value signifies a more effective design, resulting in accurate and reliable outcomes), maximize level balance (each attribute level should have an equal frequency), orthogonality (little association among various attribute levels), and minimize the measurement errors for job factors parameter estimates [11,12,21]. Despite employing experimental design methodologies, many choices may persist for presentation to participants, leading to cognitive fatigue [12]. Hence, the number of choices was kept feasible for the participants. Various experimental designs employed in the study carefully selected a smaller subset of choice scenarios, producing different D-errors and maximizing the efficiency of the statistical designs on repeated runs for constructing the choice sets [12,21]. The experimental design for the DCE was developed stepwise, which is elaborated upon in Figure 1.

Stage 1: construction of full factorial design 

Initially, a full factorial design was generated for the study [12,21]. A full factorial design examines each potential arrangement of the attribute levels. A complete factorial design enables the examination of all attribute impacts on choice, encompassing both main and interaction experimental effects. Nonetheless, in practice, the full factorial design is frequently vast and impractical, as it would be too laborious for participants to evaluate all potential combinations [12,21]. For our study, six attributes were taken with two, three, two, three, two, and two levels to generate a full factorial design, resulting in 144 choice scenarios (2^4^*3^2^) for participant presentation.

Stage 2: construction of fractional factorial design

Fractional factorial designs present each participant with only a fraction of the S-choice scenarios derived from the complete set produced by full factorial designs. Such designs minimize the profiles for which opinions are asked, as it is impractical for a single participant to go through all these decision scenarios [12,21]. In this design, subsets are chosen randomly to satisfy attribute level balance. For the study, the Ngene software (version 1.3) [21] randomly generated a fractional factorial design of 24 choice scenarios out of 144 possible choice scenarios, with different D-errors, maximizing design efficiency.

Stage 3: construction of orthogonal fractional factorial design 

Orthogonal designs condense the complete array of choices (full factorial design) into a more manageable scope (fractional factorial design). Such designs systematically pick subsets instead of randomly selecting option scenarios from the complete factorial to make the attribute levels orthogonal by removing the correlation between them [12,21]. For the study, an orthogonal fractional factorial design comprising 36 choice scenarios was generated with the lowest D-error.

Stage 4: blocking the orthogonal fractional factorial design 

In experimental design, blocking is a method employed to diminish variability and enhance result accuracy by categorizing similar experimental units. Interventions are distributed arbitrarily within each block, ensuring the intrinsic variations among the units do not obscure the treatment comparison [12,21]. The choice scenarios generated by the experimental design were later arranged into choice sets for participants to review and select their preferred job profiles. Using a block method, 36 choice scenarios were distributed in six blocks. A 6*6 block design was preferred as it was optimized, producing minimum choice situations and choice sets and less cognitive burden among the participants [12,21].

Stage 5: construction of choice sets

Choice sets consist of combinations of attribute levels depicting various job scenarios from which participants make preferences. The choice sets can be 'forced' or 'unforced'. 'Forced choice sets' only allow for two hypothetical scenarios, whereas 'unforced choice sets' would also allow the participants to select 'neither' from the given hypothetical scenarios [12,21]. For our study, choice sets for the identified attributes and levels were constructed using a pairwise design [21]. The mathematical pairing of 36 choice scenarios resulted in 90 distinct choice sets with equal probability/participation of inclusion across the six blocks. Hence, each of the six blocks comprised 15 choice sets to be presented to participants and distributed in the six DCE questionnaire versions.

## Results

Development of the DCE questionnaire

The DCE questionnaire included choice sets investigating the influence of various job attributes potentially affecting specialist physicians' preferences for accepting rural postings. The questionnaire comprised choice sets with a generic design in which two job profiles were presented as 'Job A' and 'Job B' rather than being labelled as rural or urban [10-13,22]. The job profiles consisted of a shift in the six attribute levels prescribed by the statistical design. A general opt-out choice was incorporated into the DCE questionnaire to strengthen authenticity and mitigate biases related to parameter estimates [10-13,22]. Participants were randomly distributed in six blocks and asked to choose a job posting they wished while assuming they were at the rural CHCs. The varying versions of the questionnaire were randomized across participants in the six blocks. Table 1 provides a sample version of the developed DCE questionnaire and illustrates the detailed design and structure employed in the study.

**Table 1:** Discrete choice experiment questionnaire

Choice sets (1-15)	Job 1		Job 2	
1	Workplace infrastructure	Advanced infrastructure	Workplace infrastructure	Basic infrastructure
	Salary (including rural retention bonus)	Current government salary	Salary (including rural retention bonus)	Current government salary + 50% increase in rural retention bonus
	Staffing levels and workload	Understaffed CHC with heavy workload	Staffing levels and workload	Understaffed CHC with heavy workload
	Residential facilities	No residential quarters provided but house rent allowance provided	Residential facilities	Provision of well-developed residential quarters, not free of charge
	Workplace location	150 km or more from the current place of residence or hometown (150–269 km)	Workplace location	30 km or more from the current place of residence or hometown (30–149 km)
	Transfer and promotion policies	Substantial, more rational policies based on seniority or genuine personal and medical needs along with time-bound promotions	Transfer and promotion policies	Ad-hoc policies based on current practices and norms
	Which of these two jobs do you prefer?	Job 1	Job 2	Unable to decide
2	Workplace infrastructure	Advanced infrastructure	Workplace infrastructure	Basic infrastructure
	Salary (including rural retention bonus)	Current government salary	Salary (including rural retention bonus)	Current government salary + 50% increase in rural retention bonus
	Staffing levels and workload	Understaffed CHC with heavy workload	Staffing levels and workload	Fully staffed CHC with moderate workload
	Residential facilities	No residential quarters provided but house rent allowance provided	Residential facilities	Provision of sub-standard residential quarters with limited facilities, but free of charge
	Workplace location	150 km or more from the current place of residence or hometown (150–269 km)	Workplace location	150 km or more from the current place of residence or hometown (150–269 km)
	Transfer and promotion policies	Substantial, more rational policies based on seniority or genuine personal and medical needs along with time-bound promotions	Transfer and promotion policies	Substantial, more rational policies based on seniority or genuine personal and medical needs along with time-bound promotions
	Which of these two jobs do you prefer?	Job 1	Job 2	Unable to decide
3	Workplace infrastructure	Advanced infrastructure	Workplace infrastructure	Advanced infrastructure
	Salary (including rural retention bonus)	Current government salary	Salary (including rural retention bonus)	Current government salary + 25% increase in rural retention bonus
	Staffing levels and workload	Understaffed CHC with heavy workload	Staffing levels and workload	Understaffed CHC with heavy workload
	Residential facilities	No residential quarters provided but house rent allowance provided	Residential facilities	Provision of sub-standard residential quarters with limited facilities, but free of charge
	Workplace location	150 km or more from the current place of residence or hometown (150–269 km)	Workplace location	150 km or more from the current place of residence or hometown (150–269 km)
	Transfer and promotion policies	Substantial, more rational policies based on seniority or genuine personal and medical needs along with time-bound promotions	Transfer and promotion policies	Ad-hoc policies based on current practices and norms
	Which of these two jobs do you prefer?	Job 1	Job 2	Unable to decide
4	Workplace infrastructure	Advanced infrastructure	Workplace Infrastructure	Basic infrastructure
	Salary (including rural retention bonus)	Current government salary	Salary (including rural retention bonus)	Current government salary
	Staffing levels and workload	Understaffed CHC with heavy workload	Staffing levels and workload	Fully staffed CHC with moderate workload
	Residential facilities	No residential quarters provided but house rent allowance provided	Residential facilities	Provision of sub-standard residential quarters with limited facilities, but free of charge
	Workplace location	150 km or more from the current place of residence or hometown (150–269 km)	Workplace location	30 km or more from the current place of residence or hometown (30–149 km)
	Transfer and promotion policies	Substantial, more rational policies based on seniority or genuine personal and medical needs along with time-bound promotions	Transfer and promotion policies	Substantial, more rational policies based on seniority or genuine personal and medical needs along with time-bound promotions
	Which of these two jobs do you prefer?	Job 1	Job 2	Unable to decide
5	Workplace infrastructure	Advanced infrastructure	Workplace infrastructure	Advanced infrastructure
	Salary (including rural retention bonus)	Current government salary	Salary (including rural retention bonus)	Current government salary + 25% increase in rural retention bonus
	Staffing levels and workload	Understaffed CHC with heavy workload	Staffing levels and workload	Fully staffed CHC with moderate workload
	Residential facilities	No residential quarters provided but house rent allowance provided	Residential facilities	Provision of well-developed residential quarters, not free of charge
	Workplace location	150 km or more from the current place of residence or hometown (150–269 km)	Workplace location	30 km or more from the current place of residence or hometown (30–149 km)
	Transfer and promotion policies	Substantial, more rational policies based on seniority or genuine personal and medical needs along with time-bound promotions	Transfer and promotion policies	Ad-hoc policies based on current practices and norms
	Which of these two jobs do you prefer?	Job 1	Job 2	Unable to decide
6	Workplace infrastructure	Basic infrastructure	Workplace infrastructure	Basic infrastructure
	Salary (including rural retention bonus)	Current government salary + 50% increase in rural retention bonus	Salary (including rural retention bonus)	Current government salary + 50% increase in rural retention bonus
	Staffing levels and workload	Understaffed CHC with heavy workload	Staffing levels and workload	Fully staffed CHC with moderate workload
	Residential facilities	Provision of well-developed residential quarters, not free of charge	Residential facilities	Provision of sub-standard residential quarters with limited facilities, but free of charge
	Workplace location	30 km or more from the current place of residence or hometown (30–149 km)	Workplace location	150 km or more from the current place of residence or hometown (150–269 km)
	Transfer and promotion policies	Ad-hoc policies based on current practices and norms	Transfer and promotion policies	Substantial, more rational policies based on seniority or genuine personal and medical needs along with time-bound promotions
	Which of these two jobs do you prefer?	Job 1	Job 2	Unable to decide
7	Workplace infrastructure	Basic infrastructure	Workplace Infrastructure	Advanced infrastructure
	Salary (including rural retention bonus)	Current government salary + 50% increase in rural retention bonus	Salary (including rural retention bonus)	Current government salary + 25% increase in rural retention bonus
	Staffing levels and workload	Understaffed CHC with heavy workload	Staffing levels and workload	Understaffed CHC with heavy workload
	Residential facilities	Provision of well-developed residential quarters, not free of charge	Residential facilities	Provision of sub-standard residential quarters with limited facilities, but free of charge
	Workplace location	30 km or more from the current place of residence or hometown (30–149 km)	Workplace location	150 km or more from the current place of residence or hometown (150–269 km)
	Transfer and promotion policies	Ad-hoc policies based on current practices and norms	Transfer and promotion policies	Ad-hoc policies based on current practices and norms
	Which of these two jobs do you prefer?	Job 1	Job 2	Unable to decide
8	Workplace infrastructure	Basic infrastructure	Workplace infrastructure	Basic infrastructure
	Salary (including rural retention bonus)	Current government salary + 50% increase in rural retention bonus	Salary (including rural retention bonus)	Current government salary
	Staffing levels and workload	Understaffed CHC with heavy workload	Staffing levels and workload	Fully staffed CHC with moderate workload
	Residential facilities	Provision of well-developed residential quarters, not free of charge	Residential facilities	Provision of sub-standard residential quarters with limited facilities, but free of charge
	Workplace location	30 km or more from the current place of residence or hometown (30–149 km)	Workplace location	30 km or more from the current place of residence or hometown (30–149 km)
	Transfer and promotion policies	Ad-hoc policies based on current practices and norms	Transfer and promotion policies	Substantial, more rational policies based on seniority or genuine personal and medical needs along with time-bound promotions
	Which of these two jobs do you prefer?	Job 1	Job 2	Unable to decide
9	Workplace infrastructure	Basic infrastructure	Workplace infrastructure	Advanced infrastructure
	Salary (including rural retention bonus)	Current government salary + 50% increase in rural retention bonus	Salary (including rural retention bonus)	Current government salary + 25% increase in rural retention bonus
	Staffing levels and workload	Understaffed CHC with heavy workload	Staffing levels and workload	Fully staffed CHC with moderate workload
	Residential facilities	Provision of well-developed residential quarters, not free of charge	Residential facilities	Provision of well-developed residential quarters, not free of charge
	Workplace location	30 km or more from the current place of residence or hometown (30–149 km)	Workplace location	30 km or more from the current place of residence or hometown (30–149 km)
	Transfer and promotion policies	Ad-hoc policies based on current practices and norms	Transfer and promotion policies	Ad-hoc policies based on current practices and norms
	Which of these two jobs do you prefer?	Job 1	Job 2	Unable to decide
10	Workplace infrastructure	Basic infrastructure	Workplace infrastructure	Advanced infrastructure
	Salary (including rural retention bonus)	Current government salary + 50% increase in rural retention bonus	Salary (including rural retention bonus)	Current government salary + 25% increase in rural retention bonus
	Staffing levels and workload	Fully staffed CHC with moderate workload	Staffing levels and workload	Understaffed CHC with heavy workload
	Residential facilities	No residential quarters provided but house rent allowance provided	Residential facilities	Provision of sub-standard residential quarters with limited facilities, but free of charge
	Workplace location	150 km or more from the current place of residence or hometown (150–269 km)	Workplace location	150 km or more from the current place of residence or hometown (150–269 km)
	Transfer and promotion policies	Substantial, more rational policies based on seniority or genuine personal and medical needs along with time-bound promotions	Transfer and promotion policies	Ad-hoc policies based on current practices and norms
	Which of these two jobs do you prefer?	Job 1	Job 2	Unable to decide
11	Workplace infrastructure	Basic infrastructure	Workplace Infrastructure	Basic infrastructure
	Salary (including rural retention bonus)	Current government salary + 50% increase in rural retention bonus	Salary (including rural retention bonus)	Current government salary
	Staffing levels and workload	Fully staffed CHC with moderate workload	Staffing levels and workload	Fully staffed CHC with moderate workload
	Residential facilities	No residential quarters provided but house rent allowance provided	Residential facilities	Provision of sub-standard residential quarters with limited facilities, but free of charge
	Workplace location	150 km or more from the current place of residence or hometown (150–269 km)	Workplace location	30 km or more from the current place of residence or hometown (30–149 km)
	Transfer and promotion policies	Substantial, more rational policies based on seniority or genuine personal and medical needs along with time-bound promotions	Transfer and promotion policies	Substantial, more rational policies based on seniority or genuine personal and medical needs along with time-bound promotions
	Which of these two jobs do you prefer?	Job 1	Job 2	Unable to decide
12	Workplace infrastructure	Basic infrastructure	Workplace infrastructure	Advanced infrastructure
	Salary (including rural retention bonus)	Current government salary + 50% increase in rural retention bonus	Salary (including rural retention bonus)	Current government salary + 25% increase in rural retention bonus
	Staffing levels and workload	Fully staffed CHC with moderate workload	Staffing levels and workload	Fully staffed CHC with moderate workload
	Residential facilities	No residential quarters provided but house rent allowance provided	Residential facilities	Provision of well-developed residential quarters, not free of charge
	Workplace location	150 km or more from the current place of residence or hometown (150–269 km)	Workplace location	30 km or more from the current place of residence or hometown (30–149 km)
	Transfer and promotion policies	Substantial, more rational policies based on seniority or genuine personal and medical needs along with time-bound promotions	Transfer and promotion policies	Ad-hoc policies based on current practices and norms
	Which of these two jobs do you prefer?	Job 1	Job 2	Unable to decide
13	Workplace infrastructure	Advanced infrastructure	Workplace Infrastructure	Basic infrastructure
	Salary (including rural retention bonus)	Current government salary + 25% increase in rural retention bonus	Salary (including rural retention bonus)	Current government salary
	Staffing levels and workload	Understaffed CHC with heavy workload	Staffing levels and workload	Fully staffed CHC with moderate workload
	Residential facilities	Provision of sub-standard residential quarters with limited facilities, but free of charge	Residential facilities	Provision of sub-standard residential quarters with limited facilities, but free of charge
	Workplace location	150 km or more from the current place of residence or hometown (150–269 km)	Workplace location	30 km or more from the current place of residence or hometown (30–149 km)
	Transfer and promotion policies	Ad-hoc policies based on current practices and norms	Transfer and promotion policies	Substantial, more rational policies based on seniority or genuine personal and medical needs along with time-bound promotions
	Which of these two jobs do you prefer?	Job 1	Job 2	Unable to decide
14	Workplace infrastructure	Advanced infrastructure	Workplace infrastructure	Advanced infrastructure
	Salary (including rural retention bonus)	Current government salary + 25% increase in rural retention bonus	Salary (including rural retention bonus)	Current government salary + 25% increase in rural retention bonus
	Staffing levels and workload	Understaffed CHC with heavy workload	Staffing levels and workload	Fully staffed CHC with moderate workload
	Residential facilities	Provision of sub-standard residential quarters with limited facilities, but free of charge	Residential facilities	Provision of well-developed residential quarters, not free of charge
	Workplace location	150 km or more from the current place of residence or hometown (150–269 km)	Workplace location	30 km or more from the current place of residence or hometown (30–149 km)
	Transfer and promotion policies	Ad-hoc policies based on current practices and norms	Transfer and promotion policies	Ad-hoc policies based on current practices and norms
	Which of these two jobs do you prefer?	Job 1	Job 2	Unable to decide
15	Workplace infrastructure	Basic infrastructure	Workplace infrastructure	Advanced infrastructure
	Salary (including rural retention bonus)	Current government salary	Salary (including rural retention bonus)	Current government salary + 25% increase in rural retention bonus
	Staffing levels and workload	Fully staffed CHC with moderate workload	Staffing levels and workload	Fully staffed CHC with moderate workload
	Residential facilities	Provision of sub-standard residential quarters with limited facilities, but free of charge	Residential facilities	Provision of well-developed residential quarters, not free of charge
	Workplace location	30 km or more from the current place of residence or hometown (30–149 km)	Workplace location	30 km or more from the current place of residence or hometown (30–149 km)
	Transfer and promotion policies	Substantial, more rational policies based on seniority or genuine personal and medical needs along with time-bound promotions	Transfer and promotion policies	Ad-hoc policies based on current practices and norms
	Which of these two jobs do you prefer?	Job 1	Job 2	Unable to decide

## Discussion

This study outlines the formulation of a DCE questionnaire, detailing the necessary phases for constructing choice sets through diverse experimental designs to investigate the incentive preferences of specialist physicians to enhance their retention in rural Rajasthan. A DCE is a quantitative research methodology that ascertains preferences by asking participants to select from multiple alternatives [10-13]. Each set, known as a choice set, comprises many possibilities, each characterized by various attributes and levels [10-13]. The logic for implementing this method is rooted in its ability to offer valuable insights into the job-related characteristics that specialists consider most appealing when choosing a position in rural areas [10-13].

This study utilized various effective statistical experimental designs, such as full factorial design, fractional factorial design, and orthogonal fractional factorial designs, to create choice sets for the construction of the questionnaire. Full factorial designs are a category of experimental design employed to investigate the impacts of several variables on a result. In a full factorial design, all conceivable combinations of factor levels are examined, facilitating a thorough examination of the impacts of each variable and their interrelations [12,21]. Fractional factorial designs are experimental designs employed to investigate the effects of several factors while minimizing the number of experimental runs required compared to a full factorial design. This is accomplished by evaluating only a subset of the potential combinations of factor levels [12,21]. Our study utilized orthogonal fractional factorial designs, representing a specific category of fractional factorial design utilized in experimental research. These designs are organized to preserve orthogonality, indicating that the factor levels are balanced and uncorrelated [12,21]. This facilitates the independent assessment of primary effects and specific interaction effects. These designs aimed to achieve high D-efficiency, produced the minimal standard error for the utility estimates, and ensured the creation of D-efficient choice sets for the questionnaire [10-13,21,22]. D-efficiency refers to an index utilized to evaluate and improve the statistical quality of an experimental design. Given the scarcity of literature on the detailed documentation of the DCE questionnaire development process, our study serves as a guiding tool for novice researchers, doctoral scholars, and health practitioners, equipping them with the knowledge and understanding of the steps and complexities involved in constructing various experimental designs and the development of choice sets. We aim to provide a comprehensive overview of the procedures and factors pertinent to diverse experimental designs, including full factorial, fractional factorial, and orthogonal fractional factorial designs for developing the DCE questionnaire. Each design possesses distinct advantages and disadvantages, and our analysis discusses these components, assisting researchers in constructing choice sets that align with their research objectives.

Our extensive documentation and analysis seek to provide a clear and systematic approach for generating these questionnaires, which may be adapted and employed across many research inquiries and situations. This involves explaining the rationale for choosing specific experimental designs and the process of constructing choice sets. This study also serves as a reference for individuals interested in designing DCE questionnaires, particularly those who are unfamiliar with the use and applicability of various experimental designs for assessing healthcare workers' preferences and choices that contribute to their increased retention in rural areas of low-income countries. In undertaking this, the intent is to enhance the methodological understanding of developing DCE questionnaires while offering researchers and doctoral scholars a general understanding of their utilization through a study on enhancing the retention and posting of specialist physicians in rural Rajasthan.

Despite their advantages, implementing DCEs poses various challenges. First, the speculative structure of choice sets prompted issues regarding external validity and the magnitude to which real-life judgements may align with those made by participants in experimental settings [23]. However, to enhance external validity, we designed choice sets that closely resembled real-life conditions by identifying the relevant job attributes and their levels, as informed by the in-depth interviews during the qualitative phase of the study. In addition, relevant literature reviews on HHR, expert consultations with the KIs, and pilot testing of the questionnaire confirmed the relevance of the designed choice sets to be applicable in real-life settings to inform retention strategies. Second, this approach's drawback is the level of complexity presented by the various complex choices among choice sets that include multiple attributes and levels [24]. In the study, participants might have made guesses due to the cognitive load occurring due to the presentation of multiple job profiles, thus introducing bias into the data, which might have impacted the authenticity of the results. They might have made arbitrary selections, compromising the reliability and validity of the results. However, care was taken to control such effects by keeping the number of job factors and their levels moderate. Third, the study provides valuable insights into the challenges of Rajasthan's rural healthcare system; however, rural areas in other states may face unique challenges relevant to their healthcare systems. Consequently, the results and recommendations of this study may necessitate adjustments due to the restricted generalizability of the results to cater to the specific conditions and needs of rural areas throughout different regions of India.

## Conclusions

The study describes developing a DCE questionnaire to design health policy interventions for rural retention of specialist physicians in Rajasthan, India. The process of designing the DCE questionnaire involved the creation of choice sets (hypothetical job scenarios) comprising identified attributes and levels using various experimental designs. These experimental designs included full factorial design, fractional factorial design, and orthogonal fractional factorial designs, followed by blocking of the experimental design. The designs focused on achieving high D-efficiency by minimizing the standard error for utility estimations and assuring the development of D-efficient choice sets for the questionnaire formulation. These designs incorporated the identified job attributes - workplace location, staffing level and workload, human resource policies, workplace infrastructure, residential facilities, and salary comprising rural incentives - as determined during the qualitative component of the study. The questionnaire included choice sets featuring a generic design, presenting two work profiles as 'Job A' and 'Job B'. The job profiles included a variation in the six attribute levels specified by the statistical design. A general opt-out option was integrated into the DCE questionnaire to enhance authenticity and reduce biases associated with parameter estimates. Participants were randomly allocated into six blocks and instructed to select a job posting of their preference based on the different versions of the questionnaire provided in the six blocks. However, study limitations included the hypothetical nature of choice sets, cognitive complexity resulting from the combination of different attributes and levels, and the restricted generalizability of results to rural areas outside Rajasthan, India. We hope the paper serves as a valuable introduction and guidance for novice researchers, doctoral scholars, and health practitioners interested in conducting and designing DCEs to retain specialist physicians in rural regions of LMICs and guide future policy interventions. This paper also aims to serve as a valuable introduction and guidance for those interested in conducting such experiments in HHR.
